# Mixed methods pilot evaluation of interpersonal psychotherapy for body image for adolescents

**DOI:** 10.1177/1359104520963371

**Published:** 2020-10-10

**Authors:** Fiona Duffy, Helen Sharpe, Emily Beveridge, Kate Osborne, Cathy Richards

**Affiliations:** 1 Department of Clinical Psychology, School of Health in Social Science, University of Edinburgh, Medical School, Edinburgh, UK; 2 NHS Lothian CAMHS, Royal Edinburgh Hospital, Edinburgh, UK

**Keywords:** Psychotherapy, adolescent, body image, interpersonal

## Abstract

Body dissatisfaction is common in adolescence and associated with poor outcomes. The aim of this mixed method pilot evaluation was to determine acceptability, feasibility and preliminary efficacy of Interpersonal Psychotherapy for Body Image (IPT-BI), a school-based group intervention for young people with high levels of body dissatisfaction. Eighteen participants (11–13 years, 78% female) took part in two IPT-BI groups (*n* = 10; *n* = 8). Feasibility was measured by recruitment and attrition rates; acceptability using a treatment satisfaction questionnaire and focus groups; and clinical outcomes at baseline, each session and post intervention. The majority of young people (72%, *n* = 18/25) who were referred or expressed interest went on to take part. Average session attendance was 100% and 89%. Participants expressed high levels of treatment satisfaction with 94% (*n* = 16/17) rating IPT-BI as ‘quite helpful’ or ‘very helpful’ and 94% (*n* = 16/17) stating they would recommend it to others. Preliminary exploration of efficacy showed significant improvements in body image and significant reductions in interpersonal difficulties and appearance-based conversations. Young people valued specific IPT-BI skills (role play, communication strategies), alongside generic therapeutic factors (therapeutic alliance, group cohesion). IPT-BI is feasible and acceptable with promising provisional clinical outcomes indicating the need for a fully powered randomised controlled trial.

Body image encompasses how we perceive, think and feel about our bodies. Prevalence rates of body image dissatisfaction in adolescence are high (46% of adolescent females and 26% of males) (Neumark-Sztainer et al., 2004) and body image is consistently rated by young people as one of their top concerns (YMCA, 2016). Body dissatisfaction is associated with a wide range of negative health-related outcomes, including low mood, disordered eating behaviours, smoking, risky sexual behaviours and being over or under weight (Bucchianeri et al., 2016; Schooler, 2013) highlighting that it is a serious public health concern. Consequently, there have been calls for the development and implementation of evidence-based body image interventions for young people (Mental Health Foundation, 2019).

It is well established that the interpersonal context in which a young person grows up plays an important role in the development of their body image. Ideals about appearances are communicated through mass media (films, TV, online), and also more proximally through everyday interactions with friends, family and other members of the community (Webb & Zimmer-Gembeck, 2014). Body dissatisfaction tends to be ‘concentrated’ in particular groups, such as certain friendship groups where there is greater emphasis on weight, shape and eating (Hutchinson & Rapee, 2007; Paxton et al., 1999). Young people who are vulnerable to negative body image are both more likely to choose friends who also have poor body image and become more like their friends over time (VanHuysse et al., 2016). Body dissatisfaction is more common in those who perceive greater pressure from their family and peers to be thin or look a certain way (Jones & Crawford, 2006). This perceived pressure may arise through different aspects of the ‘appearance culture’ maintained by peers and family, including participation in fat talking (self-critical comments about appearance), appearance-based teasing, comments about weight and shape, and witnessing weight control behaviours (such as dieting) in others (Jones & Crawford, 2006).

In addition to these appearance-focused interactions, there is growing evidence that the interpersonal risk for negative body image also stems from the quality of a young person’s relationships. For example, greater perceived support from peers and higher quality friendships are associated with more positive body image (Ata et al., 2007; Sharpe et al., 2014). Similarly, low perceived support, low intimacy and increasing conflict with parents is associated with the development of body dissatisfaction over adolescence (Bearman et al., 2006; May et al., 2006). Clearly, relationships are complex and those that are perceived to be supportive may also be perpetuating unhelpful messages regarding weight, shape and eating (e.g., through fat talking) and may have both positive and negative impacts on a young person’s body image. Providing young people with the tools to navigate these challenging interpersonal relationships is therefore of clinical importance.

School-based interventions can reduce body dissatisfaction among young people, with schools being an ideal setting for early intervention approaches. A systematic review by Yager and colleagues (Yager et al., 2013) found seven programmes that were effective in improving body image immediately post-intervention. However less than 20% of all programmes reviewed had sustained effects on body image at follow-up indicating further intervention development is needed, and the authors highlighted that many programmes lacked theoretical foundations for the interventions they were delivering.

Interpersonal Psychotherapy for Body Image (IPT-BI) is a school-based group intervention for young people who have high levels of body dissatisfaction. It is theoretically grounded in the model of Interpersonal Psychotherapy (Klerman & Weissman, 1993; Klerman et al., 1984), which has been shown to be effective for related difficulties in adolescence, such as depression (Mufson et al., 2011) and eating disorders (Fairburn et al., 1991; Wilfley et al., 1993). The programme is adapted from a preventative intervention for adolescent depression: Interpersonal Psychotherapy-Adolescent Skills Training (IPT-AST) (Young et al., 2016). IPT-BI has been adapted to focus specifically on body image difficulties and aims to support adolescents in developing skills to cope with complex relationships that may impact their body image, and build and make use of protective interpersonal relationships. The intervention involves one individual pre-group session followed by 8 to 10 weekly groups sessions, with 6 to 10 young people per group.

The manual and protocol for IPT-BI were developed in collaboration with young people and facilitators in an initial trial group. The aim of the current evaluation was to determine the acceptability, feasibility and initial indications of efficacy of IPT-BI based on two groups run with the finalised protocol and manual.

## Methods

### Participants

Adolescents were recruited from a local authority (public) secondary school in Scotland. Inclusion criteria were: age 11 to 18 years; English fluency; self-identified as having elevated body dissatisfaction. Exclusion criteria included meeting DSM-V criteria for an eating disorder or an intellectual disability. Participants were recruited via self-referral following promotion at school, or by referral from school staff. Young people provided informed consent and parents/caregivers were given information and ability to opt their child out of the study. Ethical approval was obtained from the University of Edinburgh and the Local Education Authority.

### Intervention

Interpersonal Psychotherapy for Body Image (IPT-BI) consists of an individual pre-group 90-minute assessment, and eight (90-minute) to ten (60-minute) weekly group sessions, allowing delivery to be pragmatically adapted based on school timetabling. Each group involves 6 to 10 pupils and is delivered within school by two facilitators.

The pre-group session focuses on rapport building and orientation to the rationale and format of the group. Body image difficulties are assessed and specific attention is paid to the past week, relating fluctuations in dissatisfaction to the interpersonal context. An interpersonal inventory is used to provide an overview of prominent relationships that have a positive or negative impact on body image. This is followed by a brief interpersonal formulation and development of treatment goals aligned with one of three IPT focal areas: disputes, transitions or sensitivities. Individual treatment goals are developed and rated pre, mid and post intervention using the Goal Based Outcome Tool (Law, 2019) (pilot goal based outcomes are available on request). Parents and/or carers were invited to a mental health and wellbeing parents evening where the group intervention was introduced and were given written information on the group content. IPT-BI is divided into three phases with the initial phase focusing on group rules and cohesion, psychoeducation and the bidirectional links between body image and interpersonal events. Communication strategies and skills are introduced, adapted from IPT-AST (Young et al., 2016) to be more aligned with interpersonal vulnerabilities in this population. This includes the introduction of a seventh rule highlighting the need for face-to-face conversations about important topics, reducing potential miscommunication via social media. These skills are taught didactically, via vignettes and role-play, prior to application of these skills to real life situations in the middle phase of the group using IPT-specific techniques including symptom review, communication analysis, role play and decision analysis. The final phase involves reviewing IPT-BI skills that have been developed, reflection on any changes in body image and interpersonal relationships and relapse prevention.

The groups took place in school with two facilitators, including the first author who is an IPT-UK accredited Clinical Psychologist and led the group intervention, and either another IPT-UK accredited Psychologist (Group 1) or a school-based Pupil Support Officer (Group 2). Co-facilitators’ main role was to support group cohesion and adherence to group rules, and in preparation were asked to read the IPT-BI manual, and meet the lead facilitator pre and post group sessions to plan and debrief.

### Quantitative measures

#### Feasibility

Feasibility measures included recruitment and attrition rates.

#### Acceptability

Acceptability was assessed using the Treatment Satisfaction Questionnaire adapted from (Mufson et al., 2015). Adolescents used Likert scales to rate how helpful the group was, whether they would recommend it to other young people, how they felt about it taking place in school, the length of each session and the overall numbers of weeks.

#### Efficacy

All outcome measures were completed at pre- and post-intervention. The primary clinical outcome was the Weight and Appearance subscales of the Body Esteem Scale for Adults and Adolescents (BES: Mendelson et al., 2001) (current sample, α = .89). Secondary outcomes were the Depression subscale of the Revised Children’s Anxiety and Depression Scale (RCADS: Chorpita et al., 2005) (α = .71), the Inventory of Interpersonal Problems-32 (IIP-32: Barkham et al., 1996) (α = .87); the Sociocultural Attitudes Towards Appearance Questionnaire (SATAQ-4: Schaefer et al., 2017) (α = .90); the Appearance Conversations with Friends (Jones et al., 2004) (α = .84) and the Eating Disorders Examination Questionnaire for Adolescents (EDE-A: Fairburn & Beglin, 1994) (α = .95), which was also used to screen young people for the presence of an eating disorder at recruitment. In addition, we collected weekly measures of body image using three items from the BES, which participants were asked to rate in relation to the past week.

### Qualitative data

Sixteen participants took part in two audio-recorded focus groups (*n* = 6 and *n* = 10) to explore views about the acceptability, perceived efficacy, and feasibility of IPT-BI. These focus groups lasted approximately 45 minutes and were conducted by three researchers who were independent of the group facilitation.

### Analyses

We examined pre-post change in outcomes using paired samples *t*-tests and calculated Cohen’s d_z_ (mean of change/SD of change). Thirteen participants (72%) had complete data; five participants were missing at least one outcome. We used pairwise deletion (reported here) and conducted sensitivity analyses using pre-intervention scores carried forward/replacement with sample mean, which produced similar results (available on request). We explored weekly change in body image using mixed effects models, with a random intercept included for participant.

Focus group transcripts were analysed using a six-step thematic analysis framework (Braun & Clarke, 2006). A deductive approach was applied to explore themes under the pre-identified areas of feasibility, acceptability and efficacy. Transcripts were read numerous times for the researchers to become familiar with the data supported by reflective notes and initial codes were created using NVivo 11. Codes were then collated and emerging themes and subthemes were developed. Two researchers examined the data independently and final themes and sub-themes were determined through discussion.

## Findings

### Quantitative data

#### Feasibility

**Figure 1.**
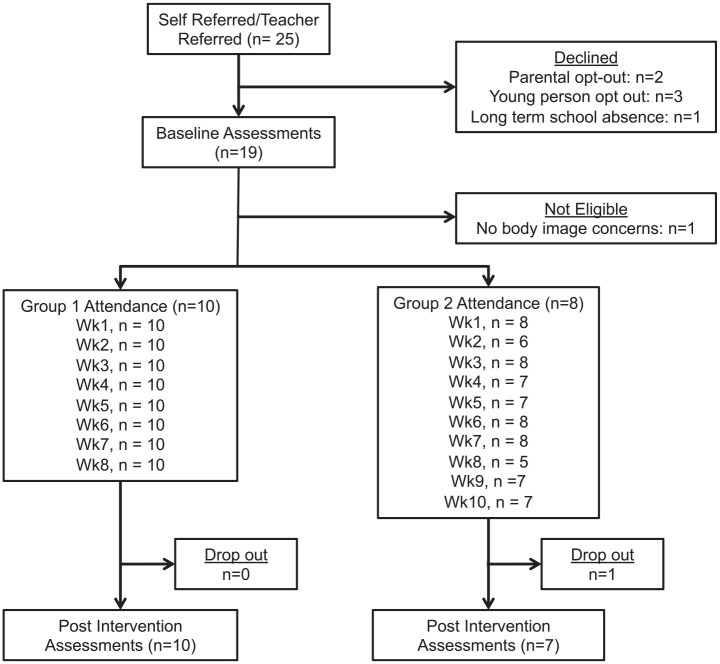
Participant flow through the trial.

Eighteen participants, recruited from 25 young people who were referred or expressed interest, took part in two groups (*n* = 10; *n* = 8) (Figure 1). Participants’ mean age was 12.42 years (SD = 0.39). Seventy-eight percent (*n* = 14) of the sample was female and 22% (*n* = 4) male. Fourteen adolescents self-identified as White (78%), one as Asian (6%), one as mixed ethnicity (6%) and two an ‘other’ ethnicity (11%).

There was no drop out from Group 1. One participant from Group 2 (6%) dropped out following week 7 and this coincided with a period of absence from school for this participant. Average session attendance was 100% in Group 1 and 89% in Group 2 (range 70–100%).

#### Acceptability

Acceptability data were available for the 17 completers. Sixteen of 17 participants (94%) rated the group as ‘quite helpful’ or ‘very helpful’, and 16 of 17 participants suggested they would recommend the group to other young people ‘quite a bit’ or ‘a lot’. All participants (*n* = 17, 100%) rated feeling either neutral or positive at the group taking place in school. The majority of participants rated the length of each sessions as being ‘about right’, (Group 1: *n* = 5, 50%; Group 2: *n* = 5, 71%) with most others rating the sessions as being ‘too short’ (Group 1: *n* = 4, 40%; Group 2: *n* = 2, 29%). In Group 1, 40% (*n* = 4) rated the overall number of sessions as being ‘about right’, and 60% (*n* = 6) as being too few. In contrast in Group 2, 57% (*n* = 4) of participants rated the overall number of sessions as being ‘about right’, and 43% (*n* = 3) as being too few.

#### Efficacy

Pre- and post-intervention outcome measures are shown in Table 1. There were significant improvements in body image from pre-intervention to post-intervention, with a medium effect size (t(16) = –2.18, *p* = 0.05, *d_z_* = 0.53). Mixed effects models exploring session-by-session change in body image showed a significant weekly rise in body image over the intervention period in both groups (Group 1, *b* = 0.09 [0.02], *p* < 0.002; Group 2, *b* = 0.13 [0.03], *p* < 0.001).

**Table 1.** Descriptive Statistics for Outcome Measures.

Construct	Pre-intervention			Post-intervention		
	*n*	Mean	*SD*	*n*	Mean	*SD*
Body image	18	1.87	0.79	17	2.26	0.81
Depressive symptoms	18	9.50	4.16	17	7.51	6.10
Interpersonal difficulties	18	43.39	16.62	17	23.53	13.40
Eating disorder symptoms	18	1.55	1.22	15	1.02	0.95
Appearance conversations	16	2.13	1.00	17	1.62	0.52
Internalisation of thin ideal	16	2.21	0.82	17	2.07	0.63

There were also significant reductions in ratings of interpersonal difficulties (t(16) = 3.34, *p* = 0.004, *d_z_* = –0.81 and appearance conversations (t(14) = 2.86, *p* = 0.01, *d_z_* = –0.74) with medium to large effects. In contrast, there were no significant changes in depressive symptoms (t(16) = 1.55, *p* = 0.14), eating disorder symptoms (t(14) = 1.82, *p* = 0.09) or internalisation of the thin ideal (t(14) = 0.84, *p* = 0.41).

### Qualitative data

Key themes from the focus groups are summarised below.

#### Feasibility

##### Theme 1: Privacy and confidentiality

When discussing the location of the IPT-BI group, all participants felt positive about it taking place in school but made suggestions about how the specific location could be improved. Group 1 had taken place in in a private meeting room, whereas Group 2 took place in a traditional classroom. This lack of privacy and confidentiality was one aspect of the intervention that Group 2 participants particularly disliked. Location was not once discussed as an issue for Group 1 participants.

*“It would be nice if it wasn’t just like in a random classroom. . . People can walk in.”* – Group 2 Participant

*“I think it should be in a more private area.”* – Group 2 Participant

##### Theme 2: Importance of timing during school day

Participants all agreed that they did not want the group taking place after school, but also highlighted educational implications associated with running the group on the same day and time each week e.g., potentially missing the same class each week.

*“I think it should be on Thursdays, block 6. ‘Cos Thursdays are terrible. I mean, uh, we miss theory home economics.”* – Group 2 Participant

##### Theme 3: Duration of intervention

The majority of participants stated that the intervention was not long enough and that they would have liked group sessions to be longer:

*“Yeah, like I think we would have all liked if it was longer ‘cos it would give us more like, more time to chat about stuff.”* – Group 1 Participant.

#### Acceptability

##### Theme 1: Supportive environment

Participants stated they enjoyed the supportive group environment and felt they benefitted from building relationships with their peers and the group facilitators. This appeared to contribute to a sense of cohesion with participants stating that having this safe place to talk about their difficulties encouraged them to share their own experiences.

*“It was also good ‘cos we were like socialising with each other and we knew that each other also had like body image issues and stuff, so it was like a chance to talk about that.”* – Group 1 Participant.

*“If you were explaining something, they [the group] were really patient and like listening properly.”* – Group 2 Participant.

*“They’re [the facilitators are] patient because they listen to what you have to say, and they’re like approachable because they’re really easy to speak to and stuff.”* – Group 1 Participant.

However, there were a minority of participants who found sharing personal experiences in a group format challenging:

*“I just don’t like it. I don’t like sharing personal stuff. It was embarrassing. It was at times. I don’t know why, it just was.”* – Group 2 Participant.

##### Theme 2: Interactive activities

Participants stated that they preferred the more interactive aspects of the intervention, particularly the role play and interactive group discussions, compared to the less interactive psycho-education.

*“Yeah I liked em you know, sharing some like personal stuff ‘cos like everyone like understood it even though you thought nobody did.”* – Group 1 Participant.

*“Role play was fun. . . It was hilarious!”* – Group 1 Participant.

##### Theme 3: Previous knowledge of cultural influences

Although most of the group material was new to young people, many participants stated they were already aware of the media’s influence on body image therefore identified this as one topic that was not found to be as enjoyable.

*“Well I found it [learning about cultural influences on body image] not really that helpful because I already knew what the internet was like and basically all of TV was like showing a bad example of what humans look like.”* – Group 2 Participant.

#### Efficacy

##### Theme 1: Developed an awareness of shared concerns

Participants described an enhanced awareness that their body image concerns were not unique and that being part of a group of individuals with similar concerns made them feel supported and understood. They stated that the group setting enabled them to work through shared concerns with other group members, provided opportunities to offer feedback and to develop and practice skills

*“I found it very helpful ‘cos then you noticed that other people are going through the exact same thing you’re going through.”* – Group 2 Participant.

*“Yeah and we kinda like realised all of us were not all like. . . we’re not like the only one who has problems like that.”* – Group 1 Participant.

##### Theme 2: Learning to identify triggering interpersonal situations

Participants identified that it was helpful learning about interpersonally triggering events that they did not have awareness of previously such as fat talk and how helpful it was to complete the weekly symptom review to support reflection on interpersonal events that had occurred that week and the impact they had had on their body image.

*“It’s [fat talk is] new to think about.” –* Group 2 Participant.

“‘*Cos we were looking back at like how you felt throughout the week you could see what was making you feel better in what weeks and what was making you feel worse in other weeks*.” – Group 1 Participant.

##### Theme 3: Improved communication with important-others

Participants reported learning skills to help cope with difficult or upsetting interactions with peers or family members, specifically learning communication strategies to resolve conflict, and manage interpersonal situations such as fat talk and appearance-based teasing.

*“I found it [communication rules and role-play] very helpful ‘cos sometimes like it can help you not escalate the situation.”* – Group 2 Participant.

*“We learned how to like, they told us how to like kinda talk to family about how we’re feeling, or like teachers and friends and stuff.”* – Group 1 Participant.

*“Like when your friends are like “oh my gosh, I look so fat” and “you’re so skinny”, “you’re like prettier than me” and stuff. How to change the subject or change how you say it.” –* Group 1 Participant.

## Discussion

IPT-BI appears to be both feasible and acceptable to young people. The recruitment strategy (using self- and teacher-referral) was successful, with only one young person who undertook baseline assessment deemed to be ineligible, and no young people meeting criteria for an eating disorder. This supports the use of this pragmatic recruitment strategy within a school environment, although some young people may be missed without a formal population screening. The most efficient recruitment approaches can be explored further when IPT-BI is implemented on a larger scale.

Satisfaction with the intervention was high, with participants rating IPT-BI as helpful and that they would recommend it to their peers. Participants identified that they particularly enjoyed learning communication strategies through group discussion and role-play and reported valuing the supportive group environment. This is aligned with a previous systematic review that highlighted the inclusion of interactive tasks in efficacious school-based body image interventions (Yager et al., 2013). Delivery in schools was acceptable to young people, although specific adaptations should be considered including where the group takes place and timetable implications. The second group involved co-delivery by a pupil support officer, indicating the potential for task-shifting in the facilitation of this group from child and adolescent mental health specialists to education staff. This is aligned with national policies promoting early intervention in a school context. Future work should investigate the potential for independent delivery by school staff within minimal training supporting a scalable intervention model.

Initial exploratory analyses indicated that IPT-BI leads to improvement in body image, although this is based on a small pilot sample. Positively, there does not appear to have been a parallel reduction in depressive symptoms, indicating that the intervention has successfully targeted body image, rather than a replication of IPT-AST (Young et al., 2006) as a preventative intervention for depression. There were also significant reductions in ratings of interpersonal difficulties and appearance conversations, which was supported by qualitative feedback where participants highlight increased awareness of interpersonally triggering situations post intervention and the benefit of learning specific IPT communication strategies. This is of considerable interest for future studies examining underpinning mechanisms of change associated with IPT-BI. However, it should be noted that participants also valued factors common to all forms of group psychotherapy, including therapeutic relationships and group cohesion, indicating the need for a controlled trial.

Limitations of this pilot include the small sample size and lack of long term follow up. Taking into account poor long term sustainable change in body dissatisfaction for similar school-based interventions (Yager et al., 2013), it will be essential that follow up is included in any future fully powered efficacy trial. A further limitation is the lack of parental involvement in the IPT-BI group protocol. IPT-AST (Young et al., 2006) includes parents during a pre- and an additional mid-group session. For pragmatic reasons, parents were not formally included within this IPT-BI pilot, and instead were invited to an optional parents evening and given group based information. The family environment is known to influence the development and maintenance of body image ideals (Jones & Crawford, 2006) and a meta-analytic review (Dowell & Ogles, 2010) has highlighted improved outcomes in child psychotherapy interventions which involve parental participation. The omission of more intensive parental involvement may have detrimental impact on short and long-term efficacy of IPT-BI and should be addressed within any future RCT.

In conclusion, IPT-BI is a theoretically-grounded, school-based intervention, which appears to be an acceptable and feasible intervention for young people with body image difficulties. A fully powered randomised control trial is warranted to now determine efficacy including the potential to investigate task-shifting to school-based professionals to support large scale dissemination.
